# What determines the direction of subliminal priming

**DOI:** 10.2478/v10053-008-0024-1

**Published:** 2008-07-15

**Authors:** Piotr Jaśkowski, Rolf Verleger

**Affiliations:** 1 Department of Cognitive Psychology, University of Finance and Management, Warszawa, Poland; 2 Department of Neurology, University of Luebeck, Germany

**Keywords:** subliminal priming, inverse and straight priming, backward masking

## Abstract

Masked stimuli (primes) can affect the preparation of a motor response to subsequently presented target stimuli. Reactions to the target can be facilitated (straight priming) or inhibited (inverse priming) when preceded by a compatible prime (calling for the same response) and also when preceded by an incompatible prime. Several hypotheses are currently under debate. These are the self-inhibition (SI) hypothesis, the object-updating (OU) hypothesis, and mask-triggered inhibition (MTI) hypothesis. All assume that the initial activation of the motor response is elicited by the prime according to its identity. This activation inevitably leads to straight priming in some cases and the mechanisms involved are undisputed. The hypotheses differ, however, as to why inverse priming occurs. The self-inhibition (SI) hypothesis assumes that the motor activation elicited by a prime is automatically followed by an inhibition phase, leading to inverse priming if three conditions are fulfilled: perceptual evidence for the prime has to be sufficiently strong, it has to be immediately removed by the mask, and the delay between the prime and target has to be long enough for inhibition to become effective. The object-updating (OU) hypothesis assumes that inverse priming is triggered by the mask, provided that it contains features calling for the alternative response (i.e. the one contrasting with the response induced by the prime). The MTI hypothesis assumes that the inhibitory phase is triggered by each successive stimulus which does not support the perceptual hypothesis provided by the prime. Based mostly on our own experiments, we argue that (1) attempts to manipulate the three factors required by the SI hypothesis imply changes of other variables and that (2) indeed, other variables seem to affect priming: prime-mask perceptual interaction and temporal position of the mask. These observations are in favor of the MTI hypothesis. A limiting factor for all three hypotheses is that inverse priming is larger for arrows than for other shapes, making it doubtful as to what extent the majority of studies on inverse priming, due to their use of arrows, can be generalized to other stimuli.

## INTRODUCTION

### Subliminal priming

Research on unconscious influences on behavior has been attracting much interest in recent years because these kinds of studies give some insight into what conscious awareness is for. Indeed, by comparing what the human brain can do without bothering for conscious awareness to those situations where conscious mediation is needed, we can try to infer the role of the latter process.

To make stimuli unseen and still effective on behavior, usually backward masking is used in which a subsequent stimulus (the mask) is able to reduce visibility of the preceding stimulus (the prime). With the appropriate timing and spatial arrangement of masks and primes, this technique works very effectively with a wide range of stimuli. A currently wide-spread hypothesis assumes that the mask disrupts the reentry process (an iterative loop comparing sensory input with stored representations), which is thought to be necessary for creating a vivid percept (Di Lollo & Enns, 2000).

Even if completely masked, primes have been shown to be processed quite effectively. Such processing has been demonstrated by showing that masked stimuli can affect responses to or categorizations of a target presented after the prime and mask. This method is called subliminal priming. With this method, it was shown that primes can affect detection (Fehrer & Raab, 1962), pattern recognition (Neumann & Klotz, 1994), recognition of word meaning (Draine & Greenwald, 1998), and categorization (Dehaene, Naccache, Cohen, Le Bihan, Mangin, Poline, & Riviére, 2001; Kiefer & Spitzer, 2000). Although it is still debatable which level of processing the subliminal primes can really affect (Abrams & Greenwald, 2000; Breitmeyer, Ro, & Singhal, 2004; Breitmeyer, Öğmen, Ramon, & Chen, 2005; Kunde, Kiesel, & Hoffmann, 2003), there are currently no doubts that people’s behavior may depend on sensory information they are unaware of.

Subliminal priming of motor reactions was demonstrated for the first time by Fehrer and Raab (1962) . They measured simple reaction times (RT) to presentations of a square. With onset asynchronies ranging from 0 to 75 ms, two other squares were displayed left and right of the original one, masking the priming square by metacontrast (Breitmeyer, 1984) and thereby reducing its perceived brightness to an extent depending on the interval between the prime and mask, with a maximal reduction for the onset asynchrony of 75 ms. Because of the well-known inverse dependence between simple reaction time and brightness (e.g. Bartlett & MacLeod, 1954; Jaśkowski, 1985; Mansfield, 1973), Fehrer and Raab expected the longest RTs for 75 ms. It turned out, however, that RT did not depend on perceived brightness at all. Fehrer and Raab suggested that the primes triggered reactions before their perceived brightness was reduced by the mask (Note 1).

This effect was further explored by Neumann and Klotz (1994; see also Klotz & Neumann, 1999). In one of their experiments, two well visible shapes were presented left and right of fixation. One shape was the target, requiring a left or right key-press depending on its presentation side. The priming figures were small replicas of those used in the visible pair and were completely masked by the main figures through metacontrast. The primes were indeed not noticeable, as was checked in a separate session. If decision making had relied only on conscious recognition of the stimuli, the unseen prime should not have affected participants’ RTs. In fact, responses to the target stimuli were speeded up by compatible and delayed by incompatible primes, that is, it did matter whether the small copy of the target shape in the priming pair was on the same side as in the visible pair or on the other side to the visible pair. Priming with such an outcome will be referred to as “straight priming”, following Verleger et al. (Verleger, Jaśkowski, Aydemir, Van der Lubbe, & Groen, 2004).

This finding was replicated in numerous studies performed in Neumann’s (Ansorge, Klotz, & Neumann, 1998; Fellows, Tabaza, Heumann, Klotz, Neumann, Schwarz, Noth, & Töpper, 2002; Klotz & Neumann, 1999; Klotz & Wolff, 1995) as well as in other laboratories (Jaśkowski, Van der Lubbe, Schlotterbeck, & Verleger, 2002; Jaśkowski, Skalska, & Verleger, 2003; Jaśkowski et al., 2002; Leuthold & Kopp, 1998; Mattler, 2003).

### Direct parameter specification

 To explain their findings, Neumann and Klotz (1994) applied Neumann’s (1990) theory of direct parameter specification (DPS). According to the most recent version of this theory (see Ansorge & Neumann, 2005) the fate of the information which, due to masking, did not reach the level of consciousness depends on participants’ current intentions. They search the environment for information that helps to perform the task. For example, in the case of choice responses with left and right hands, what has to be specified on the bases of incoming stimuli is the response hand. Other parameters could already be specified before the stimulus was presented. Therefore, any stimulus that appears is evaluated for the missing task-relevant information. In a similar vein, Kiesel et al. (Kiesel, Kunde, & Hoffmann, this wolume) assumed that subliminally presented stimuli can trigger responses to the extent they fit so-called action triggers (i.e. action release conditions) which are specified off-line by the demands of a task to be done. 

According to these theories, conscious awareness is not necessary to specify the free parameters or to compare the stimulus features with the action triggers. Rather, unconsciously processed information, as in experiments with subliminal priming, is sufficient. This means that consciousness plays only the role of an agent that has to determine what to do, and has to control whether everything goes well (Jaśkowski et al., 2003), whereas actual task performance is delegated to automatic unconscious processes.

This view was corroborated by electrophysiological data (Jaśkowski et al., 2002; Jaśkowski et al., 2003; Leuthold & Kopp, 1998). Using the original Neumann and Klotz paradigm, Leuthold and Kopp (1998) showed that the negativity contralateral to the responding hand (lateralized readiness potential, LRP), indicating selection and execution of the response to the target, is preceded by a smaller positive wave in incompatible trials and by a negative wave (partially overlapping with the target-related LRP) in compatible trials. These small waves were interpreted as reflections of prime-related activations automatically elicited according to the prime identity and participants’ task.

### Inverse priming

The DPS theory suggests that what is formed consciously in a choice task is an intention. Those stimuli encompassed by the intention can then be identified automatically, without mediation of consciousness, and can trigger response activation. Therefore, if some prime, visible or not, is incompatible with the target, the wrong response is initially activated. When the target appears, the ongoing motor activity has to be canceled and replaced by the preparation of the alternative response.

This picture was remarkably complicated by Eimer and Schlaghecken’s (1998) findings. Unlike the majority of studies cited above, Eimer and Schlaghecken used pattern-masking rather than metacontrast. Three shapes – a prime, a mask and a target – were consecutively displayed at fixation. The prime and the target were double arrow-heads, pointing to the left or to the right. Participants had to respond with their left or right hands depending on whether the arrows pointed left or right. The mask was formed from the two target shapes overlaid on one another.

The pattern of results was different from that obtained with metacontrast masking: the RTs were shorter and more accurate when the priming and target arrows pointed in different directions (incompatible trials) than when they pointed in the same directions. Throughout this article we will refer to this phenomenon as “inverse priming”.

The LRPs obtained in Eimer and Schlaghecken’s experiment looked different from those reported by Leuthold and Kopp (1998) . In the case of the compatible trials, the response-related negative LRP was preceded by two smaller deflections, a negative one observed around 240 ms after the prime, followed by a positive one at 360 ms. In the case of the incompatible trials, the polarities of the two waves preceding the target-related negativity were reversed: first a positive wave appeared and then a negative one, which overlapped with the target-related negativity.

## SELF-INHIBITION

In the light of the theories at the time, Eimer and Schlaghecken’s (1998) outcome was unexpected. To account for their results, they proposed that the initial prime-induced activation of some response is replaced by inhibition. In more detail, when the temporal interval between prime and target is short, subliminal primes activate responses in accordance with the DPS theory. This is because the target appears while prime-induced activation still persists. This phase of activation is then followed by an inhibitory phase which becomes effective if the distance between the mask and target becomes long enough. Then, if a target identical or similar to the prime appears (compatible trial), the proper hand is inhibited, leading to delayed responding. The reversed situation occurs with incompatible trials. This idea was consistent also with the observed LRP: according to these authors, the two waves preceding the target-related negativity reflect the excitatory and inhibitory phases of the prime-induced response activation.

### The effect of spatial and temporal variations on priming

This simple model was shown to successfully account for a number of results collected subsequently by Eimer and Schlaghecken. First of all, it was shown that the priming effect critically depends on the temporal interval between the prime and target: straight priming occurred only for short prime-target intervals while inverse priming appeared once the interval was long enough.

This finding suggested a perfect congruence between Eimer and Schlaghecken’s new priming effect and earlier findings. Indeed, in those studies, the primes were masked by metacontrast, and so the mask simultaneously used to play the role of the target. Therefore, for efficient masking of the prime, the prime-target interval had to be quite short (about 50 ms). Moreover, masking is known to be more efficient when stimuli are presented peripherally. Therefore, primes and masks were usually presented left and right of fixation. With this, conditions were favorable for straight priming to occur (see below). To exclude the possibility that the differences between Eimer and Schlaghecken’s (1998) and earlier results were due to the type of masking, Eimer (1999) performed an experiment with metacontrast masking and showed that, again, priming became inverse when the prime-target interval was sufficiently long. It should, however, be noted that in order to freely manipulate the prime-target interval without affecting the prime visibility, a mask had to be inserted between the prime and target. Therefore, the stimulating sequence consisted of three consecutive stimuli, as in Eimer and Schlaghecken’s original study. 

### Prime visibility and strength of sensory representation

 To account for further results, Schlaghecken and Eimer (2002) had to introduce another assumption, namely that an important factor determining the sign of the priming effect is the visibility of the prime. First of all, inverse priming had never been noted when the prime was left unmasked (Klapp & Hinkley, 2002; Verleger et al., 2004). Moreover, Eimer and Schlaghecken (2002) showed that the priming effect increased from negative (inverse priming) to positive values (straight priming) when masking efficiency decreased. The masks were composed of tilted lines of different lengths and orientations. Mask efficiency was manipulated by changing the number of line elements or the prime duration. The transition point between inverse and straight priming occurred precisely when *d*’ started to diverge from zero, suggesting an important role of the prime’s visibility. At that time, these authors concluded: “These results suggest that the conscious awareness of a prime stimulus and the presence or absence of response inhibition [reflecting inverse priming] in subliminal priming are linked.” (Eimer & Schlaghecken, 2002, p. 520). However, in light of further evidence the claim was dismissed that there existed a simple and uniformly effective connection between conscious visibility of the prime and straight priming on the one hand, and invisibility of the prime and inverse priming on the other (see Schlaghecken, Rowley, Sembi, Simmons, & Whitcomb, this volume; Sumner, this volume; for the reasons see also below). 

### Strength of sensory representation

In a subsequent series of experiments, Schlaghecken and Eimer (2002) manipulated the temporal interval between the prime and mask (while keeping the mask-target distance constant), and showed that for short intervals the priming effect was straight and turned to inverse for longer intervals. To account for this finding, they assumed that the extension of the interval gave time for increasing the prime-evoked sensory representation and that this sensory representation had to exceed some limit in order to evoke inverse priming. To support their view, they conducted another experiment in which the prime was presented against a random-dot background supposed to degrade the prime and thereby to reduce its sensory strength. They expected inverse priming for intact primes and straight priming for degraded ones. In fact, priming was inverse for the intact prime but was remarkably reduced for the degraded masks.

Therefore, the original inhibition hypothesis by Eimer and Schlaghecken (1998) needed to be supplemented. Schlaghecken and Eimer (Bowman, Schlaghecken, & Eimer, 2006; Schlaghecken & Eimer, 2002) formulated a more elaborated model in which the inhibitory phase of the basic mechanism (activation followed by inhibition) was triggered only if the prime-evoked sensory input was, first, strong enough, and second, immediately erased by the mask. Therefore, this model can be summarized as follows: (1) a prime that resembles a target activates the motor response required by the target, (2) this activation is automatically self-inhibited provided that the strength of the prime’s sensory representation is sufficiently large to trigger this inhibitory mechanism, and (3) self-inhibition can only prevail when perceptual evidence for the prime is immediately removed (Eimer & Schlaghecken, 2002); (see Note 2). This latter feature is responsible for why the sign of the priming effect and prime visibility are linked, as is suggested by the above-quoted citation from Eimer and Schlaghecken’s (2002) study. In a similar vein, Klapp and Hinkley (2002) assumed that under conditions of low prime visibility, unconscious processes, which are generally inhibitory in nature, win the competition over conscious processes, which are excitatory.

One might consider these assumptions to be contradictory because, on the one hand, the prime must be strongly perceived, and on the other hand, it must be effectively removed from being perceived. In any case, these two antagonistic mechanisms might account for widely differing patterns of results, and thus these hypotheses might prove difficult to confute. The situation seems quite well-defined when the turning point from inverse to straight priming is strictly linked to visibility, as had been done by Eimer and Schlaghecken (2002) . In such a case, inverse priming is expected for *d*’ = 0 and straight priming occurs for *d*’ > 0. Therefore, the hypothesis would seem to be falsified once inverse priming occurs for visible primes (*d*’ > 0) or straight priming for *d*’ = 0. Such results have indeed been presented (Jaśkowski & Przekoracka-Krawczyk, 2005; Lleras & Enns, 2004; Verleger et al., 2004). More recently, however, the linkage between inhibition and prime visibility has been relaxed by the adherents of the SI hypothesis. Consequently, any refutation of the SI hypothesis becomes extremely difficult because whatever the sign of the priming effect, it may be explained by the SI hypothesis by assuming that the strength of sensory representation either crossed the inhibition threshold or did not. Indeed, inverse priming with clearly suprathreshold primes has not been taken as an argument against the SI hypothesis (Experiment 1 of Schlaghecken, Rowley, Sembi, Simmons, & Whitcomb, this volume).

**Figure 1.**
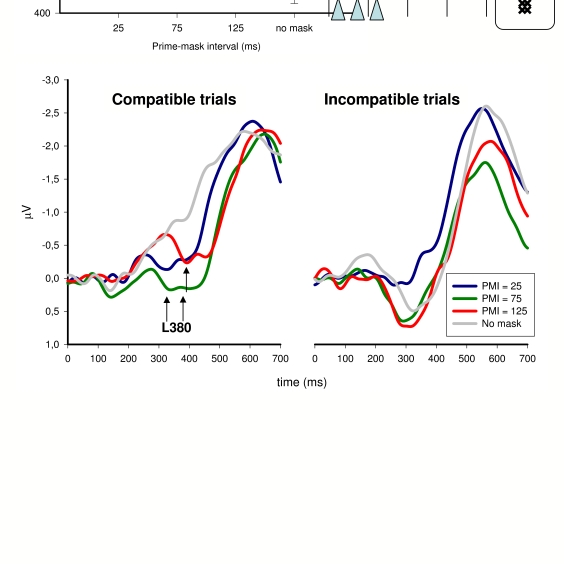
Results obtained by Jaśkowski et al. (in press). The primes were two identical double arrows presented above and below fixation. They were followed by a distractor being formed from two overlaid arrows and presented at fixation. The targets were also double arrows presented to the left and right of fixation. The course of a trial is presented in the upper-right diagram. Thd triangles represent possible temporal positions of the distractor. Reaction times are presented in the upper-left graph. LRPs (separately for the compatible and incompatible trials) are presented in the lower row. The arrows indicate the positions of a deflection called L380, which reflects the (mask-triggered) inhibitory phase.

However, we showed (Jaśkowski, 2007; Jaśkowski, Białuńska, Tomanek, & Verleger, in press) that inverse priming may appear even if primes are not occluded by the mask. Rather it might be sufficient that the mask is not ignored. In one of this series of experiments (Jaśkowski et al., in press), arrow primes were presented 2° above and below fixation, and arrow targets were presented 2° left and right of fixation. The mask consisted of overlaid primes, but this “mask” was presented at fixation, and thus did not mask the primes at all. Prime-“mask”-SOAs (SOA = stimulus onset asynchrony) were 25, 75, 125 ms in random order, while the prime-target-SOA was always 205 ms. In a control condition, no mask was presented at all. Prime effects were inverse when prime-“mask”-SOA was 75 ms, equal to zero for prime-“mask”-SOA = 125 ms, and straight for the no-mask condition. LRPs displayed a triphasic shape for the compatible trials and a biphasic shape for incompatible trials, similar to Eimer and Schlaghecken’s (1998) results in the “normal” masking situation. Moreover, a clear dependence of LRP on SOA was found, suggesting that the longer the SOA was, the later the second wave of the triphasic complex appeared (see Fig. 1). In our view, this experiment casts serious doubts on the role that is assigned in the SI hypothesis to the mask. Indeed, in the above-described experiment the mask did not remove the perceptual evidence for the prime. Nevertheless, occurrence of the inhibition phase of the LRP is strictly related to the moment of mask presentation. This may be taken to suggest that a more critical factor than occlusion from visibility is just the presentation of a temporally trailing stimulus at the same place or in the nearest vicinity.

## PRIME-MASK INTERACTION

### Mask structure matters

The size of inverse priming (Eimer & Schlaghecken, 1998) has continuously shrunk over the years, from about -50 ms in 1998 (Eimer & Schlaghecken, 1998; Experiment 1) via about -20 ms in 2002 (Eimer & Schlaghecken, 2002) to about -10 ms in recent articles (Schlaghecken & Eimer, 2006; Experiment 1). Had Eimer and Schlaghecken published this small effect in 1998, perhaps neither we nor hardly anybody else would have cared about it. But since the 1998 paper started with that spectacular effect, several authors were attracted by this provocative phenomenon and a fruitful scientific discussion was started.

Eimer and Schlaghecken’s (1998) approach was generalized by Klapp and Hinkley (2002) to encompass the difference between conscious and unconscious processing. These two papers were criticized by Lleras and Enns (2004) and Verleger et al. (2004) , who underscored that the masks in these two papers were composed of target-like figures, or at least contained features the participants searched for in the target stimuli (Note 3) to properly perform the task. Following this observation, Lleras and Enns (2004) suggested that each new stimulus is integrated with an already existing scene and an updated version of that scene is created. If the scene has been changed, the old version is replaced by a new version. When the new elements of the scene call for the other behavior than that already initiated by the prime, participants change their behavior accordingly. This means that if elements are found which call for another response, the prime-triggered activation is stopped and activation of the alternative response is initiated. Therefore, with respect to the priming phenomenon, the most crucial assumption of this hypothesis is that only the new elements of the scene trigger the updating routine and start a possible correction of behavior.

Lleras and Enns’ (2004) object-updating (OU) hypothesis was supported by the finding that masks composed of elements irrelevant to the task (e.g. vertical and horizontal lines) lead to straight priming, while inverse priming was obtained only for masks containing objects which shared features with the targets (Jaśkowski & Przekoracka-Krawczyk, 2005; Lleras & Enns, 2004; Verleger et al., 2004).

### What is updated in the scene?

**Figure 2.**
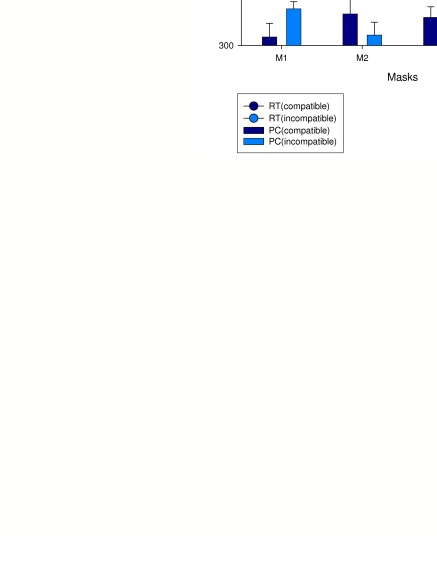
Results of an experiment by Jaśkowski and Przekoracka-Kraw-czyk (2005). The primes were double arrows presented at fixation. They were masked by four different masks shown in the middle row. The upper graph presents prime identifications for the four masks. Reaction time (RT) and proportion correct (PC) obtained in this experiment are presented in the lower graph.

An important question to be asked is what exactly is updated in the scene when a mask appears. The answer seems easy in the case of Eimer and Schlaghecken’s original masks composed of two double-arrows. Indeed, in those experiments the mask replaced the prime. Therefore, the only new element added with the presentation of the mask was the arrow pointing in the opposite direction. However, Jaśkowski and Przekoracka-Krawczyk (2005) demonstrated inverse priming with masks which were formed from some arrows randomly distributed over an area (Fig. 2). What about scene updating in this case? One can assume that an object is more abstract than just a shape at a given location. Therefore, it is conceivable that (i) the prime-compatible part of the mask does not call for an update even if the shapes presented in the masks shift their locations in respect to the prime or, even more, that (ii) participants’ motor behavior is updated only if stimuli assigned to different responses appear. With this extension of the OU hypothesis, one can easily explain Jaśkowski and Przekoracka-Krawczyk’s (2005) inverse priming. Indeed, although there are some new arrows pointing in the same direction as the prime, one can assume that only those which point in the other direction call for a routine which corrects the behavior (i.e. starts the alternative response and/or inhibits the prime-triggered response).

**Figure 3.**
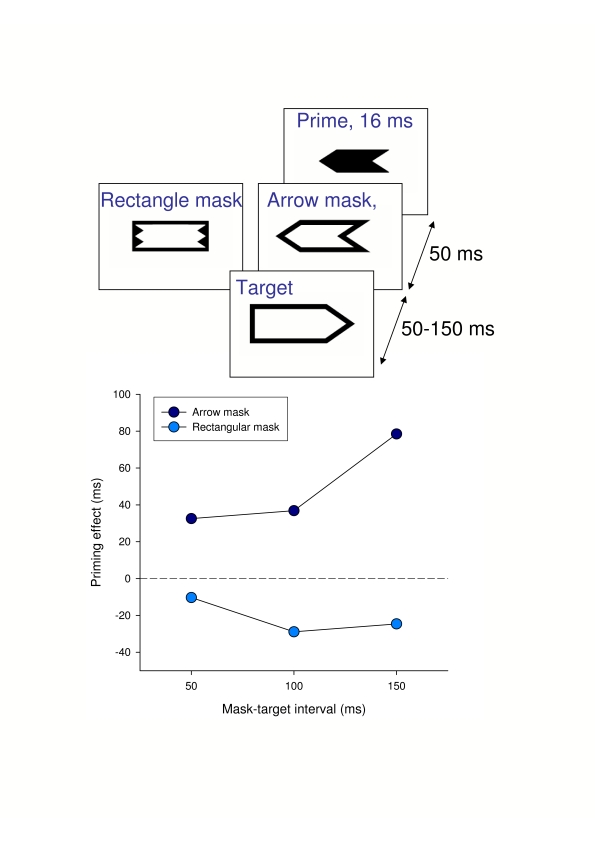
Results of an unpublished experiment (Jaśkowski & Trzcińska, unpublished results). An arrow prime presented at fixation was followed by a mask being an outline of a rectangle or of an arrow. The targets were still larger arrows. Priming effect [= RT(incompatible) – RT(compatible)] as a function of the prime-target interval is presented in the graph.

Does this mean that new prime-like arrows that point in the same direction as the prime do not affect the response at all? A simple experiment (Jaśkowski & Trzcińska, unpublished results) provides convincing evidence that this is not true. Arrows were used as primes (Fig. 3). The prime was followed by a mask which was either the outline of a slightly larger arrow pointing in the same direction as the prime, or a rectangle. The masks masked the prime by meta-contrast. Still larger arrows were used as targets. The arrow mask should not have called for the updating routine, not providing any new information on motor behavior. Therefore, the priming effect was expected to be the same for the arrow and rectangle masks. In fact, large straight priming was observed in the case of the arrow mask as if the activation induced by prime and mask summed up (for a similar effect see also Jaśkowski et al., 2003). In the case of the rectangle mask, inverse priming was noted (Note 4). Therefore, to maintain the OU hypothesis, one has to assume that all new elements are updated, but the elements pointing in the other direction to the prime are more important. In other words, in masks containing features of both primes to an equal extent, elements similar to the actually presented prime will be less salient, therefore, elements similar to the opposite prime will act as a second prime in the opposite direction.

Experiments using random-line masks, and arrows as primes and targets have been interpreted as supporting the OU hypothesis, but were in fact inconclusive. The reason is that it is unclear whether random-line masks are relevant or not in the context of arrow primes and targets. Likewise, it is unclear whether random-line masks are or are not similar to arrow primes and targets. On the one hand, proponents of the OU hypothesis considered random-line masks as being relevant and as being similar to arrow primes and targets (Lleras & Enns, 2004). Because of this task relevance of the masks, an object updating would be required in the case when an arrow prime preceded a random-line mask. Thus, the observed inverse priming effect under random-line mask conditions was considered to support the OU hypothesis.

On the other hand, however, proponents of the SI hypothesis considered random-line masks as being task-irrelevant and dissimilar to the prime and target arrows, meaning that the inverse priming effect under random-line mask conditions provided evidence for an independence of inverse priming from task relevance of the masks (or for an independence of inverse priming from similarity between masks and prime or target). Evidently, this alternative interpretation of the results would be in stark contrast with the predictions of the OU hypothesis. As there is no independent evidence to decide whether random-line masks are relevant or not (and whether they are similar or not to the prime and target arrows), the inverse priming effect with random-line masks cannot be considered as evidence for or against the OU hypothesis.

## THE ROLE OF TASK-RELEVANT FEATURES IN THE MASK

The OU hypothesis does not explain instances of inverse priming by arrow primes when the mask contains vertical and horizontal lines only (Klapp & Haas, 2005; Lleras & Enns, 2004; Schlaghecken & Eimer, 2006; Schlaghecken et al., this volume; Verleger, Görgen, & Jaśkowski, 2005), inverse priming of bar primes masked by arrow masks (Verleger et al., 2005), nor inverse priming of square vs. diamond primes in Eimer (1999) , where the octagonal mask lay inside each prime, its corners rotated by 22.5° with respect to the corners of either prime. These instances of inverse priming require an alternative explanation, preferably one that covers all instances of inverse priming.

Therefore, we proposed an alternative hypothesis called “mask-triggered inhibition” (Jaśkowski, 2007; Jaśkowski & Przekoracka-Krawczyk, 2005) that may be considered as a synthesis of both the discussed hypotheses. First, in contrast to Verleger et al. (2004) and Verleger, Ewers, and Jaśkowski (submitted) , we concede that inhibition of the primed response does occur. The main difference from Schlaghecken and Eimer’s view (Bowman et al., 2006; Schlaghecken & Eimer, 2002, 2006) is that this inhibition is considered to be evoked by the mask rather than being a rigid consequence of prime activation occluded from further perceptual evidence. More specifically, we assume that each immediately following stimulus appearing within the focus of attention, which does not support the perceptual hypothesis concerning the prime’s identity (Note 5), will inhibit the ongoing action and thereby activate the alternative response (Note 6). However, the mask produces this inhibition particularly if and insofar as it contains elements similar to the primes: Perception of any such elements informs the system that activation was premature and should be inhibited. This assumption is farther away from the OU hypothesis than it may appear at first sight: Elements of the mask are actually not needed in the present hypothesis for substituting the prime by a mask (working virtually as a new prime), but rather are assumed to get selected by top-down control because observers cannot ignore task-relevant elements, and therefore lead to inhibition of any activation.

In one of Jaśkowski’s (2007) recent experiments, the primes were again presented above and below fixation and the “mask” at fixation, but the mask did not consist of diagonal lines anymore, rather it looked like a cross and a square. This slight change from the above-mentioned critical experiment (see Fig. 1) where the overlaid-prime non-masking “mask” was used, abolished inverse priming (there was straight priming with all SOAs, at least up to +20 ms), in agreement with the proposal that the mask produces inhibition particularly if it contains elements similar to the primes. Nevertheless, even this irrelevant mask modified priming effects, depending on the prime-mask and mask-target SOAs. This finding is inconsistent with predictions of the SI and OU hypotheses as (i) the “mask” acted as a flanker rather than a mask, therefore, the changes in the priming effect cannot be assigned to removing the sensory evidence by the mask as the SI hypothesis assumes; (ii) the flanker/“mask” has no relevant features which call for the updating routine, an important assumption of the OU hypothesis.

According to this view, spatial and temporal conditions affecting the perception of the mask are no less important for inverse priming to occur than they are in their effect on the primes. Thus, presenting the prime and mask at fixation is a favorable condition for inverse priming (Lingnau & Vorberg, 2005; Schlaghecken & Eimer, 2000, 2002), possibly not only because the primes are more poorly perceived in the periphery than at fixation, but also because the same is true for the masks, which when presented in the periphery, the perceptual system is not confronted as intensively with interfering mask elements as it is when the mask is presented at fixation or with larger size (Lingnau & Vorberg, 2005).

Thus, we propose that the mask acts as a “false friend” to the processing system. It is a friend by preventing the system from misperceiving the primes as targets. But it is a false friend by presenting features that might be misunderstood by the system as being relevant. As a safe-guard against this interference, existing response activations get inhibited, putting them at a disadvantage for the upcoming response to the target.

These findings also forced Lleras and Enns (2006) to revise their OU hypothesis. In that recent article, the OU hypothesis was supplemented with two additional assumptions, one of which was very close to our mask-triggered inhibition. The only difference is that they did not assume that mask-feature relevance as such is important. Instead, they maintain that it is important if new elements of the scene call for updating.

The second assumption they added was called the repeated location advantage. It refers to the observation that inverse priming is more likely when the prime and target are presented at the same or some nearby location. Lleras and Enns (2005) showed that when the prime and target were presented at the same position, inverse priming occurred also for irrelevant masks (consisting of vertical and horizontal lines). In contrast, when only the prime and mask were presented at the same position, while the target was displayed aside, inverse priming occurred only for the relevant mask. Lleras and Enns (2006) argued that the visual system considers a spatiotemporally proximal prime and target as two instantiations of a single object which changes/develops in time.

To further support this new version of the OU hypothesis (we will refer to it as OU+) they performed an experiment where the basic predictions of the hypothesis were tested. Several conditions were compared. The prime (double arrow) was always presented at fixation. The target (double arrow) was presented either on fixation (i.e. at fixation) or off fixation (i.e. either above or below fixation). Moreover, the conditions differed as to where the mask (overlaid double arrow or randomly distributed vertical and horizontal lines) was placed. In the flashed mask condition the mask was displayed at fixation, covering the prime, in the flashed flankers conditions two identical masks were presented as flankers left and right of fixation, and in the continuous flankers conditions the two flankers remained on the screen until participants responded. Consistent with the OU+ hypothesis, prime/target/mask similarity, abrupt onset of flankers, and “spatial similarity”, that is, the proximity of the presented objects, made the priming effect more negative.

The OU hypothesis (like the other two) develops “by budding”: once a problem is encountered, a new assumption is added. At the same time, no clear evidence has been provided that enhancement of inverse priming with relevant masks is due to object updating. Above we argued that the MTI hypothesis provides an alternative explanation.

## OCCURRENCE OF INVERSE PRIMING WITH STIMULI OTHER THAN ARROWS

**Figure 4.**
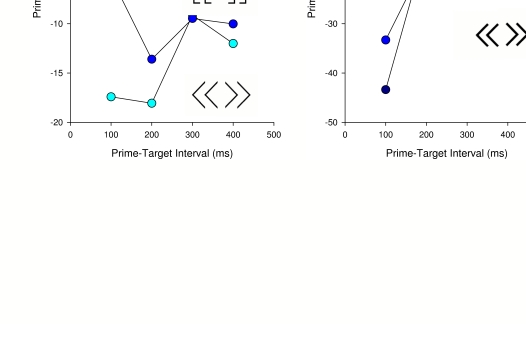
Results of two experiments by Jaśkowski and Ślósarek (2007) with primes of different shapes. Priming effect [= RT(incompatible) – RT(compatible)] is plotted as a function of the prime-target interval. The shapes of the primes used are shown near each plot. In the experiment whose results are presented in the left graph, the masks were formed from lines of different orientation and length, randomly dispersed over an area. The mask used in the other experiment was formed from the two primes of a given pair overlaid with one another. Note that overlaying the two pairs of the primes forms the identical mask.

 The majority of studies reviewed so far used arrow-head lines as primes and targets. Thus, the question arises as to how much inverse priming actually occurs with other stimuli. In fact, inverse priming has been obtained with a number of different stimuli: inverted arrows (Eimer & Schlaghecken, 1998), squares vs. diamonds (Eimer, 1999; Mattler, 2005), and “bars” (Verleger et al., 2005). Directly comparing arrows to letters (H and S, which are the standard stimuli applied in many studies on the interfering effect of flanking stimuli), Verleger et al. (submitted) obtained inverse priming with arrows only. They did obtain a differential effect of masks on letter priming, very similar to the effect they obtained with arrows: Priming was less straight with the overlaid-primes than with the random-line mask. But the effect remained in the positive range. Another interesting result was obtained by Verleger et al. (2005) : When masked by their overlaid-primes mask, “bars” (a horizontal line with a response-relevant vertical line at its left or right side) tended to have a straight priming effect. It was only when they were masked by overlaid arrows that inverse priming occurred. To account for these two unclear effects, Verleger et al. (submitted) presumed that arrows are special in evoking inverse priming, possibly related to their stronger interfering effects as irrelevant flankers (Mattler, 2003; Wascher, Reinhard, Wauschkuhn, & Verleger, 1999), and to their potency to activate pre-motor cortex by default (Praamstra, Boutsen, & Humphreys, 2005; Verleger, Vollmer, Wauschkuhn, & Wascher, 2000). To investigate these matters, Jaśkowski and Ślósarek (2007) compared the priming effects of different shapes as primes and targets, holding the mask constant (random-lines mask). In agreement with the presumed special role of arrows, inverse priming evoked by arrows was larger than by brackets and by diamond vs. square (Fig. 4, upper panel). However, Jaśkowski and Ślósarek reasoned that the decisive factor might again be the prime-mask interaction (i.e. the ease with which the prime and target features could be singled out in the mask structure). In support of this assumption, diamond vs. cross, which stimuli can be singled out with ease from an overlaid-arrows mask but which clearly do not possess any overlearned directional feature, had a strong inverse priming effect, no less than arrows at 

100 ms mask-target SOA (though less at longer SOAs; see Fig. 4 lower panel). In conclusion, while some aspects of differences in priming effects between stimuli are still unclear, some of these effects may be accounted for by the prime-mask interaction, thus by the same factor that was considered to be relevant above in discussing the variation of inverse priming induced by different masks.

## Notes

1 Note that unlike contemporary demonstrations of the priming effect, Fehrer and Raab have used masked targets rather than masked primes.

2 It seems that an additional necessary assumption is that under non-masking but not backward masking conditions, perceptual evidence for primes continues in iconic memory. Otherwise, a removal of the prime coincides with the end of that stimulus and inverse priming should appear also under non-masking conditions.

3 Unlike Eimer and Schlaghecken (1998) , Klapp and Hinkley (2002) used a mask composed of letters: WXXW and XWWX. Still, one may speculate that they contained the searched-for features (arrows). Both these letters (especially X) might be perceived as a compound of two opposing arrows.

4 This experiment was motivated by an experiment reported recently by Dirk Vorberg (2005). Unlike our experiment, he used a mask that was a rectangle with an arrow-like cut-out. This cut-out matched exactly to the priming arrow.

5 Without this assumption it is impossible to account for Jaśkowski and Trzcińska’s experiment described above within the MTI hypothesis. So, this is a post-hoc assumption which was not presented in the previous formulations of the hypothesis (Jaśkowski, 2007; Jaśkowski & Przekoracka-Krawczyk, 2005).

6 Results of numerous recent studies (Burle, Bonnet, Vidal, Possamai, & Hasbroucq, 2002; Praamstra & Seiss, 2005; Vidal, Grapperon, Bonnet, & Hasbroucq, 2003) demonstrate that in choice tasks, activation of a response is associated with inhibition of the alternative response.

## References

[R1] Abrams R. L., Greenwald A. G. (2000). Parts outweigh the whole (word) in unconscious analysis of
						meaning.. Psychological Science.

[R2] Ansorge U., Klotz W., Neumann O. (1998). Manual and verbal responses to completely masked (unreportable)
						stimuli: Exploring some conditions for the metacontrast
						dissociation.. Perception.

[R3] Ansorge U., Neumann O. (2005). Intentions determine the effect of invisible metacontrast-masked
						primes: evidence for top-down contingencies in a peripheral cuing
						task.. Journal of Experimental Psychology: Human Perception and
						Performance.

[R4] Bartlett N. R., MacLeod S. (1954). Effect of flash and field luminance upon human reaction
						time.. Journal of Optical Society of America.

[R5] Bowman H., Schlaghecken F., Eimer M. (2006). A neural network model of inhibitory processes and cognitive
						control.. Visual Cognition.

[R6] Breitmeyer B. G. (1984). Visual masking: An integrative approach..

[R7] Breitmeyer B. G., Öğmen H., Ramon J., Chen J. (2005). Unconscious and conscious priming by forms and their
						parts.. Visual Cognition.

[R8] Breitmeyer B. G., Ro T., Singhal N. S. (2004). Unconscious color priming occurs at stimulus- not
						percept-dependent levels of processing.. Psychological Science.

[R9] Burle B., Bonnet M., Vidal F., Possamai C.-A., Hasbroucq T. (2002). A transcranial magnetic stimulation study of information
						processing in the motor cortex: Relationship between the silent period and
						the reaction time delay.. Psychophysiology.

[R10] Dehaene S., Naccache L., Cohen L., Le Bihan D., Mangin J.-F., Poline J.-B., Riviére D. (2001). Cerebral mechanisms of word masking and unconscious repetition
						priming.. Nature Neuroscience.

[R11] Di Lollo V., Enns J. T. (2000). Competition for consciousness among visual events: The
						psychophysics of reentrant visual processes.. Journal of Experimental Psychology: General.

[R12] Draine S. C., Greenwald A. G. (1998). Replicable unconscious semantic priming.. Journal of Experimental Psychology: General.

[R13] Eimer M. (1999). Facilitatory and inhibitory effects of masked prime stimuli on
						motor activation and behavioural performance.. Acta Psychologica.

[R14] Eimer M., Schlaghecken F. (1998). Effects of masked stimuli on motor activation: Behavioral and
						electrophysiological evidence.. Journal of Experimental Psychology: Human Perception and
						Performance.

[R15] Eimer M., Schlaghecken F. (2002). Links between conscious awareness and response inhibition:
						Evidence from masked priming.. Psychonomic Bulletin & Review.

[R16] Fehrer E., Raab D. (1962). Reaction time to stimuli masked by metacontrast.. Journal of Experimental Psychology.

[R17] Fellows S., Tabaza R., Heumann M., Klotz W., Neumann O., Schwarz M., Noth J., Töpper R. (2002). Modification of a functional motor task by not consciously
						perceived sensory stimuli.. NeuroReport.

[R18] Jaśkowski P. (1985). The effect of visual adaptation on simple motor reaction time.
						Part I.. Studia Psychologica.

[R19] Jaśkowski P. (2007). The effect of nonmasking distractors on the priming of motor
						responses.. Journal of Experimental Psychology: Human Perception and
						Performance.

[R20] Jaśkowski P., Białuńska A., Tomanek M., Verleger R. Mask- and distractor-triggered inhibitory processes in the
						priming of motor responses. An EEG study.. Psychophysiology.

[R21] Jaśkowski P., Przekoracka-Krawczyk A. (2005). On the role of mask structure in subliminal
						priming.. Acta Neurobiologiae Experimentalis.

[R22] Jaśkowski P., Skalska B., Verleger R. (2003). How the self controls its “automatic pilot”
						when processing subliminal information.. Journal of Cognitive Neuroscience.

[R23] Jaśkowski P., Ślósarek M. (2007). How important is prime’s gestalt for subliminal
						priming?. Consciousness and Cognition.

[R24] Jaśkowski P., van der Lubbe R., Schlotterbeck E., Verleger R. (2002). Traces left on visual selective attention by stimuli that are not
						consciously identified.. Psychological Science.

[R25] Kiefer M., Spitzer M. (2000). Time course of conscious and unconscoius semantic brain
						activation.. NeuroReport.

[R26] Kiesel A., Kunde W., Hoffmann J. (2007). Mechanisms of subliminal response priming.. Advances in Cognitive Psychology.

[R27] Klapp S. T., Haas B. W. (2005). Nonconscious influence of masked stimuli on response selection is
						limited to concrete stimulus-response associations.. Journal of Experimental Psychology: Human Perception and
						Performance.

[R28] Klapp S. T., Hinkley L. B. (2002). The negative compatibility effect: Unconscious inhibition
						influences reaction time and response selection.. Journal of Experimental Psychology: General.

[R29] Klotz W., Neumann O. (1999). Motor activation without conscious discrimination in metacontrast
						masking.. Journal of Experimental Psychology: Human Perception and
						Performance.

[R30] Klotz W., Wolff P. (1995). The effect of a masked stimulus on the response to the masking
						stimulus.. Psychological Research.

[R31] Kunde W., Kiesel A., Hoffmann J. (2003). Conscious control over the content of unconscious
						cognition.. Cognition.

[R32] Leuthold H., Kopp B. (1998). Mechanisms of priming by masked stimuli: Inferences from
						event-related brain potentials.. Psychological Science.

[R33] Lingnau A., Vorberg D. (2005). The time course of response inhibition in masked
						priming.. Perception & Psychophysics.

[R34] Lleras A., Enns J. T. (2004). Negative compatibility or object updating? A cautionary tale of
						mask-dependent priming.. Journal of Experimental Psychology: General.

[R35] Lleras A., Enns J. T. (2005). Updating a cautionary tale of masked priming: A reply to Klapp
						(2005).. Journal of Experimental Psychology: General.

[R36] Lleras A., Enns J. T. (2006). How much like a target can a mask be? Geometric, spatial, and
						temporal similarity in priming. A reply to Schlaghecken and Eimer
						(2006).. Journal of Experimental Psychology: General.

[R37] Mansfield R. J. W. (1973). Latency function in human vision.. Vision Research.

[R38] Mattler U. (2003). Priming of mental operation by masked stimuli.. Perception & Psychophysics.

[R39] Mattler U. (2005). Inhibition and decay of motor and nonmotor
						priming.. Perception & Psychophysics.

[R40] Neumann O. (1990). Direct parameter specification and the concept of
						perception.. Psychological Research.

[R41] Neumann O., Klotz W., Umiltá C., Moscovitch M. (1994). Motor responses to nonreportable, masked stimuli: Where is the
						limit of direct parameter specification?. Conscious and unconscious information processing.

[R42] Praamstra P., Boutsen L., Humphreys G. W. (2005). Frontoparietal control of spatial attention and motor intention
						in human EEG.. Journal of Neurophysiology.

[R43] Praamstra P., Seiss E. (2005). The neurophysiology of response competition: Motor cortex
						activation and inhibition following subliminal response
						priming.. Journal of Cognitive Neuroscience.

[R44] Schlaghecken F., Eimer M. (2000). A central-peripheral asymmetry in masked priming.. Perception & Psychophysics.

[R45] Schlaghecken F., Eimer M. (2002). Motor activation with and without inhibition: Evidence for a
						threshold mechanism in motor control.. Perception & Psychophysics.

[R46] Schlaghecken F., Eimer M. (2006). Active masks and active inhibition: A comment on
								Lleras and Enns (2004) and on Verleger, Jaśkowski Aydemir, van der Lubbe and Groen (2004). Active masks and active inhibition: A comment on Lleras and Enns
						(2004) and on Verleger, Jaśkowski, Aydemir, van der Lubbe, and
						Groen (2004).. Journal of Experimental Psychology: General.

[R47] Schlaghecken F., Rowley L., Sembi S., Simmons R., Whitcomb D. (2007). The negative compatibility effect: A case for
						self-inhibition.. Advances in Cognitive Psychology.

[R48] Sumner P. (2007). Negative and positive masked-priming – implications
						for motor inhibition.. Advances in Cognitive Psychology.

[R49] Verleger R., Ewers T., Jaśkowski P. On the special role of arrow-masked arrows as flankers: Inverse
						priming depends on types of stimuli and of masks..

[R50] Verleger R., Görgen S., Jaśkowski P. (2005). An ERP indicator of processing relevant gestalts in masked
						priming.. Psychophysiology.

[R51] Verleger R., Jaśkowski P., Aydemir A., Van der Lubbe R. H. J., Groen M. (2004). Qualitative differences between conscious and non-conscious
						processing? On negative and positive priming effects induced by masked
						arrows.. Journal of Experimental Psychology: General.

[R52] Verleger R., Vollmer C., Wauschkuhn B., Wascher E. (2000). Dimensional overlap between arrows as cueing stimuli and
						responses? Evidence from contra-ipsilateral differences in EEG
						potentials.. Cognitive Brain Research.

[R53] Vidal F., Grapperon J., Bonnet M., Hasbroucq T. (2003). The nature of unilateral motor commands in between-hand choice
						tasks as revealed by surface Laplacian estimation.. Psychophysiology.

[R53a] Vorberg D. (2005). Ist Hemung oder Bahnung die Grundlage des
						umgekehrten Priming-Effekte?.

[R54] Wascher E., Reinhard M., Wauschkuhn B., Verleger R. (1999). Spatial S-R compatibity with centrally presented stimuli: An
						event-related asymmetry study on dimensional overlap.. Journal of Cognitive Neuroscience.

